# Thioredoxin Reductase as a Target for Antibacterial Gold Compounds in *Burkholderia cenocepacia*: Disclosing the Molecular Basis of Enzyme Inhibition

**DOI:** 10.1002/cbic.70462

**Published:** 2026-07-10

**Authors:** Stefano Zineddu, José Aleixo de Azevedo‐França, Martina Aguanno, Valentina Pecchioli, Giarita Ferraro, Virginia Cuomo, Antonello Merlino, Luigi Messori

**Affiliations:** ^1^ Laboratorio Metalli in Medicina Dipartimento di Chimica “Ugo Schiff” – DICUS Università degli Studi di Firenze Sesto Fiorentino Florence Italy; ^2^ Dipartimento di Scienze Chimiche Università degli Studi di Napoli Federico II, Complesso Universitario di Monte Sant’Angelo Napoli Italy

**Keywords:** antibacterial activity, gold(I) compounds, mass spectrometry, metallodrugs, thioredoxin reductase

## Abstract

Thioredoxin reductase from *Burkholderia cenocepacia* (Bc‐TrxR) is a recognized intracellular target of antibacterial gold(I) compounds, yet the molecular basis of enzyme inhibition remains unclear. Here, we report an integrated structural, biophysical, and biochemical investigation of recombinant Bc‐TrxR and its interaction with three prototypical gold(I) agents: auranofin (**AF**) and two trimethylphosphine‐thiolate derivatives (**Au1** and **Au2**), previously identified as potent enzyme inhibitors. The enzyme was expressed, purified, and its crystal structure solved at 2.52 Å resolution, revealing a homodimeric architecture closely resembling that of the *Escherichia coli* enzyme. Each subunit contains FAD‐ and NADPH‐binding domains and a catalytic Cys–Cys motif representing a plausible coordination site for gold fragments. High‐resolution ESI‐MS provided direct molecular evidence for the formation of defined gold‐protein adducts, with up to two Au(I) centres bound per subunit. The observed mass shifts are consistent with selective coordination of gold fragments to catalytically relevant cysteine residues, effectively blocking redox turnover. Together, crystallographic and mass spectrometric results disclose the molecular mechanism of Bc‐TrxR inhibition by phosphine‐thiolate gold(I) complexes and establish a structural framework for the rational design of next‐generation antibacterial metallodrugs.

## Graphical abstract

The molecular basis of thioredoxin reductase inhibition by antibacterial gold(I) compounds is disclosed. X‐ray crystallography and high‐resolution ESI‐MS reveal selective gold coordination to catalytically relevant cysteine residues of *Bc*‐TrxR, leading to stable protein–gold adducts and disruption of enzyme redox activity. These findings provide a structural framework for the design of new antibacterial metallodrugs.

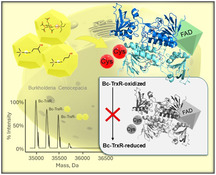

## 1 Introduction

Antibiotic resistance is one of the most pressing challenges in modern medicine. The rapid emergence and global spread of multidrug‐resistant bacteria threaten decades of therapeutic progress and are projected to cause up to 10 million deaths a year by 2050 [1]. Inappropriate antibiotic use in healthcare and agriculture has significantly accelerated this phenomenon. The Centres For Disease Control and Prevention estimated that around 29% of the 236.4 million antibiotic prescriptions issued by doctors and in emergency departments in 2022 were unnecessary [2]. This promotes the selection of resistant strains through multiple mechanisms, including: (i) target modification, (ii) reduced drug accumulation, (iii) enzymatic inactivation, and (iv) biofilm formation [3, 4].

Among problematic Gram‐negative pathogens, a member of the *Burkholderia cepacia* complex poses a particular threat to patients with cystic fibrosis, especially those with weak immune systems [5]. The opportunistic bacterium, *Burkholderia cenocepacia*, exhibits intrinsic and acquired resistance to multiple antibiotic classes and can cause persistent respiratory infections and often‐fatal septicemia in vulnerable patients [6].

In light of these considerations, new strategies are needed to combat antibiotic‐resistant bacteria. In this context, metal‐based compounds have emerged as promising candidates owing to their unique chemical versatility and distinct mechanisms of action [7, 8]. Gold(I) complexes, in particular, have demonstrated potent antibacterial activity, especially towards Gram‐positive bacteria. Several studies have demonstrated that many Gram‐positive bacteria rely almost entirely on the thioredoxin system to maintain intracellular redox homeostasis, lacking efficient glutathione‐based backup pathways. Consequently, inhibition of TrxR results in severe oxidative stress and rapid bacterial death [9, 10]. This mechanistic vulnerability largely explains the remarkable susceptibility of Gram‐positive pathogens, including multidrug‐resistant strains, to auranofin (an FDA‐approved gold(I) drug originally developed for the treatment of rheumatoid arthritis) and related gold(I) compounds [11, 12]. These compounds can be toxic to bacteria through the cationic metallic center itself or the organic or inorganic scaffold that stabilizes the metal center. Gold‐based compounds are among the most promising in this diverse group of molecules.

Particular interest has been directed toward bacterial thioredoxin reductase (TrxR) as a therapeutic target for overcoming antimicrobial resistance. The potent antibacterial activity of these agents has therefore been directly linked to their ability to selectively target the thioredoxin system. This highlights bacterial TrxR as an attractive target for the development of novel metallodrugs capable of circumventing conventional resistance mechanisms.

Recent studies have demonstrated that auranofin and related gold(I) phosphine‐thiolate complexes strongly inhibit *B. cenocepacia* TrxR (Bc‐TrxR). However, the molecular and structural basis of this inhibition is not well understood [13]. Thus, we conducted further research to reveal the molecular processes leading to enzyme inhibition.

**FIGURE 1:**
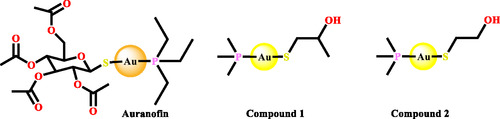
Molecular structures of the investigated gold(I) compounds: auranofin and the phosphine‐thiolate derivatives **Au1** and **Au2**.

Based on the above reasons, this report details the expression in *Escherichia coli* and purification of recombinant Bc‐TrxR, as well as the determination of its crystal structure. Independent electrospray ionization mass spectrometry (ESI‐MS) experiments complement the characterization of its solution behaviour. ESI‐MS measurements were subsequently used to investigate the interactions between Bc‐TrxR and some representative gold(I) compounds in solution (see Figure 1). Our results provide direct evidence of gold(I) coordination with the active‐site cysteine residues. These findings lend weight to the notion of a cysteine‐targeting mechanism for enzyme inhibition and offer a structural framework for designing enhanced antimicrobial gold agents.

## 2 Results and Discussion

### 2.1 Production, Purification, and Characterization of *B. Cenocepacia* TrxR

**FIGURE 2:**
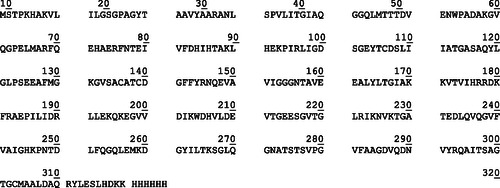
Primary sequence of Bc‐TrxR.

Recombinant Bc‐TrxR was successfully expressed in *E. coli* and purified through a combination of Ni^2+^‐affinity and size‐exclusion chromatography. The primary sequence of the protein is shown in Figure 2. Nonspecifically bound proteins were removed during the washing phase of the affinity chromatography step prior to application of the imidazole gradient. The unbound fraction was collected and analysed to confirm the absence of Bc‐TrxR. Upon application of a linear imidazole gradient (20–500 mM), the target protein eluted as a single, well‐defined chromatographic peak corresponding to fractions 12–25 (Figure S1), indicating efficient capture and recovery of recombinant Bc‐TrxR. sodium dodecyl sulphate polyacrylamide gel electrophoresis (SDS‐PAGE) analysis of these fractions revealed a predominant band at ≈35 kDa, consistent with the expected molecular weight of the monomeric enzyme, with only minor contaminating species detectable (Figure S2). Subsequent size‐exclusion chromatography yielded a single, symmetric peak (Figure S3), consistent with a homogeneous protein preparation. SDS‐PAGE analysis of the collected fractions confirmed the high purity of the sample, with fractions 10–12 showing a dominant Bc‐TrxR band and being selected for further studies (Figure S4). Analysis of soluble and insoluble fractions demonstrated that Bc‐TrxR is present exclusively in the soluble fraction, while no protein band corresponding to Bc‐TrxR was detected in the wash unbound sample.

**FIGURE 3:**
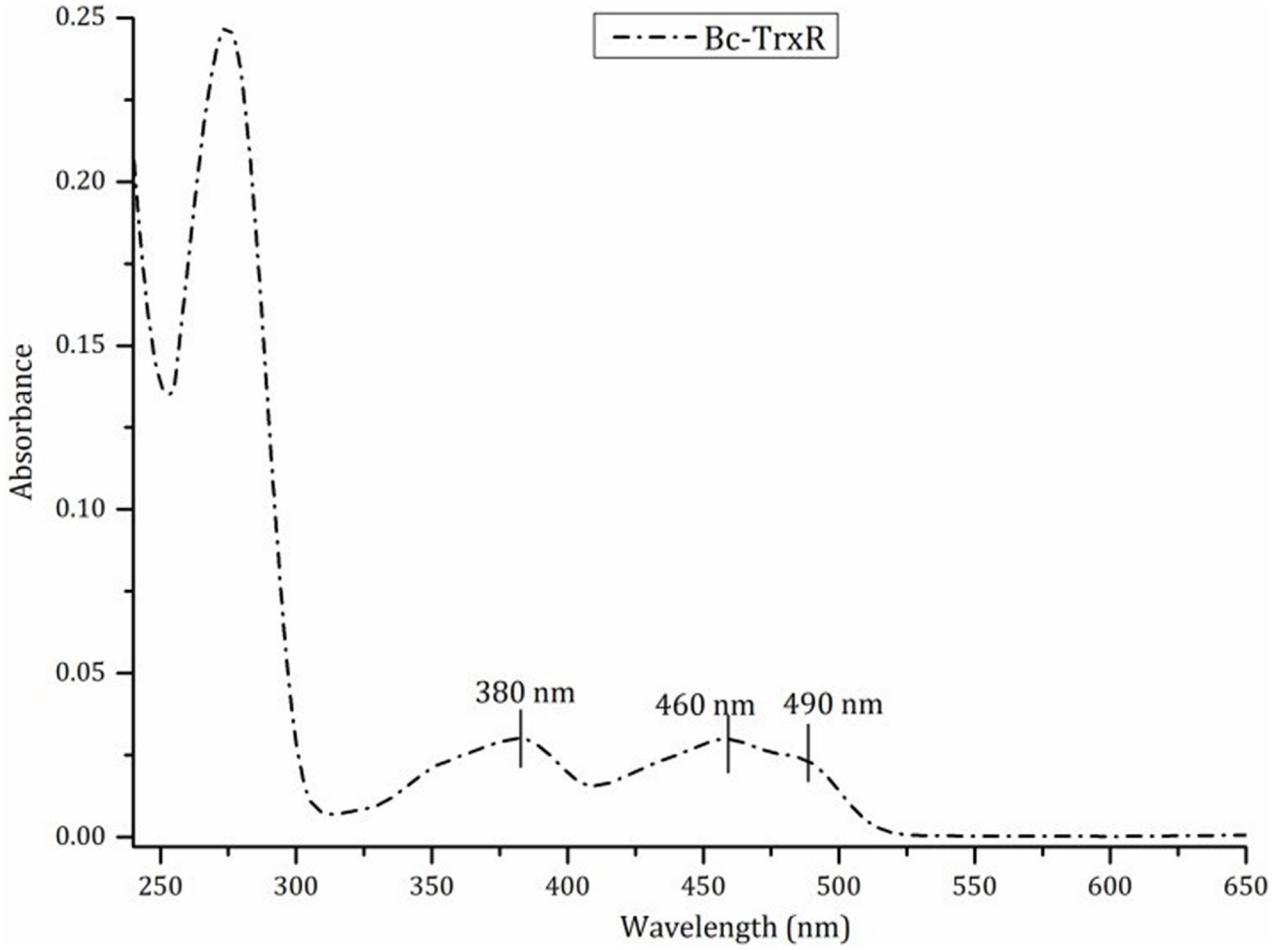
UV–vis absorption spectrum of Bc‐TrxR collected at 8.5 µM concentration in 20 mM Tris‐HCl at pH 7.5.

The UV–visible absorption spectrum of the purified enzyme shows the characteristic flavoprotein bands attributable to protein‐bound flavin adenine dinucleotide (FAD), with maxima located at 380 and 460 nm (with a shoulder at 490 nm) corresponding to π → π* transitions in the isoalloxazine ring. This finding confirms correct incorporation of the FAD cofactor (Figure 3).

**FIGURE 4:**
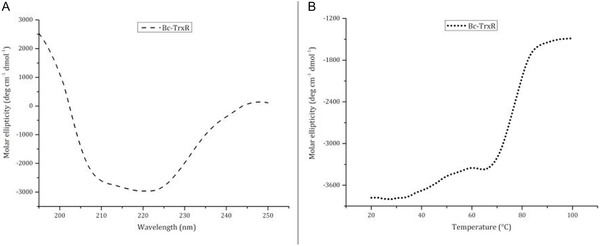
Far UV‐CD spectrum of Bc‐TrxR collected at 4.3 µM concentration in 20 mM Tris‐HCl at pH 7.5 at 20°C (panel A). Bc‐TrxR thermal denaturation profile (panel B); CD signal was monitored at 222 nm as a function of temperature.

Far‐UV circular dichroism (CD) measurements reveal that the protein has a well‐defined secondary structure (Figure 4A). The CD spectrum shows a profile typical of a protein with a relatively high α‐helix content, featuring two broad minima at 210 and 222 nm, as well as a positive peak around 195 nm. This profile is consistent with the canonical α/β fold of bacterial low‐molecular‐weight TrxRs (40.7% α‐helix, 15% β‐sheet, 44.3% loop/coil/turns). To verify the effect of the temperature on the protein structure, the CD signal at 222 nm was followed at increasing temperature from 20°C to 100°C. The denaturation curve (Figure 4B) indicates that the protein undergoes irreversible cooperative unfolding with a melting temperature of 77.0 ± 1.0°C. This value agrees with literature data reporting high stability of TrxR from *E. coli* (Ec‐TrxR), which remains stable for more than 1 h at 80°C [14], although Tm of Bc‐TrxR is higher than that reported for Ec‐TrxR (66°C) [15].

### 2.2 Crystal Structure of Bc‐TrxR

**FIGURE 5:**
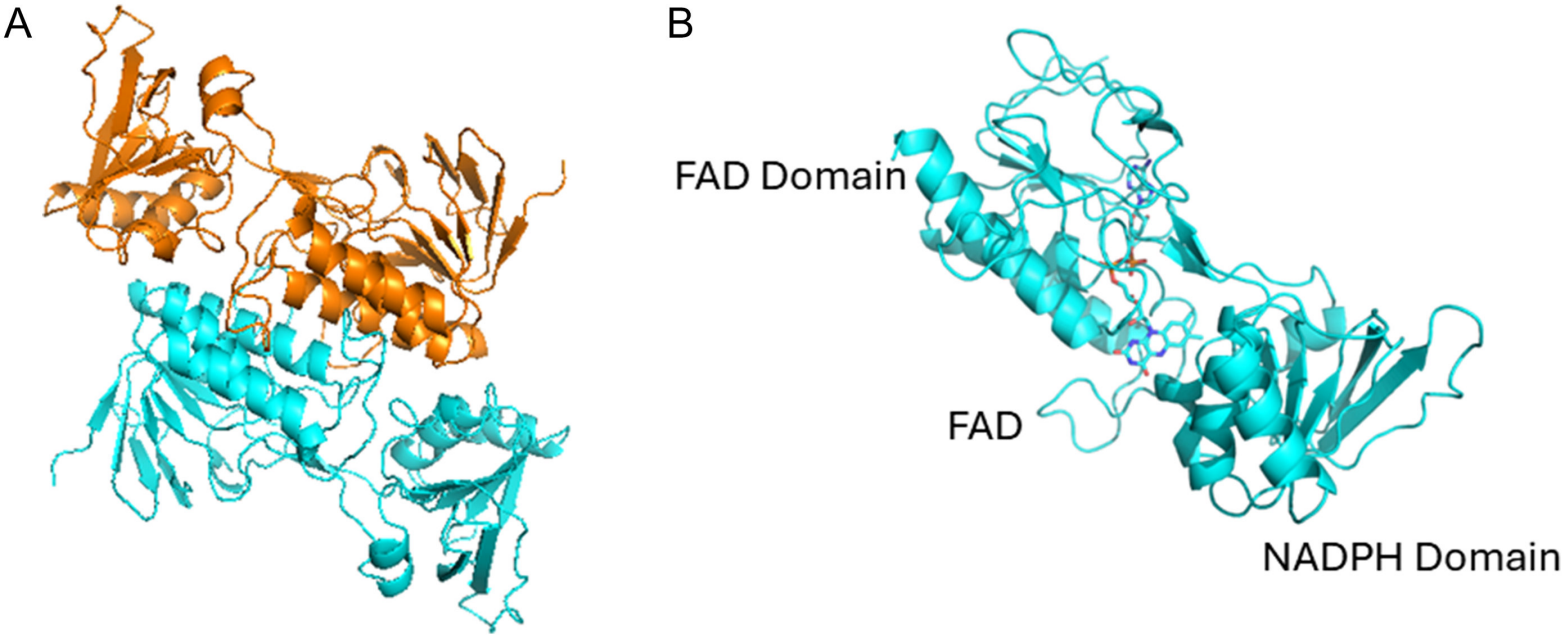
Overall structure of Bc‐TrxR homodimeric assembly (panel A, chains in orange and cyan) and of the Bc‐TrxR subunit (panel B, cyan). FAD and NADPH domains and the FAD binding site are highlighted in panel (B).

The structure of Bc‐TrxR was determined by the molecular replacement method, using the coordinates of the *E. coli* enzyme (Protein Data Bank (PDB) code 1TDE without ligands [16], 68% sequence identity) as a starting model (see Methods and Table S1 for details). The crystal structure refines at 2.52 Å resolution to R‐factor and R_free_ values of 26.0 and 36.6%, respectively, and contains one subunit in the asymmetric molecule. As expected, Bc‐TrxR is a homodimeric protein (Figure 5A), with each subunit formed by two domains: the FAD domain and the reduced nicotinamide adenine dinucleotide phosphate (NADPH) domain (Figure 5B). In Bc‐TrxR, the two domains have the same relative orientation observed in the *E. coli* enzyme in each subunit. The dimeric assembly is also similar to that of oxidized Ec‐TrxR reported in the PDB under the accession code 1TDE [17]. Root mean square deviation between the carbon alpha atoms of the two enzymes is as low as 0.84 Å. Other structural parameters that were evaluated for the two enzymes and reported in Table S2, highlight the similarity between the two structures.

**FIGURE 6:**
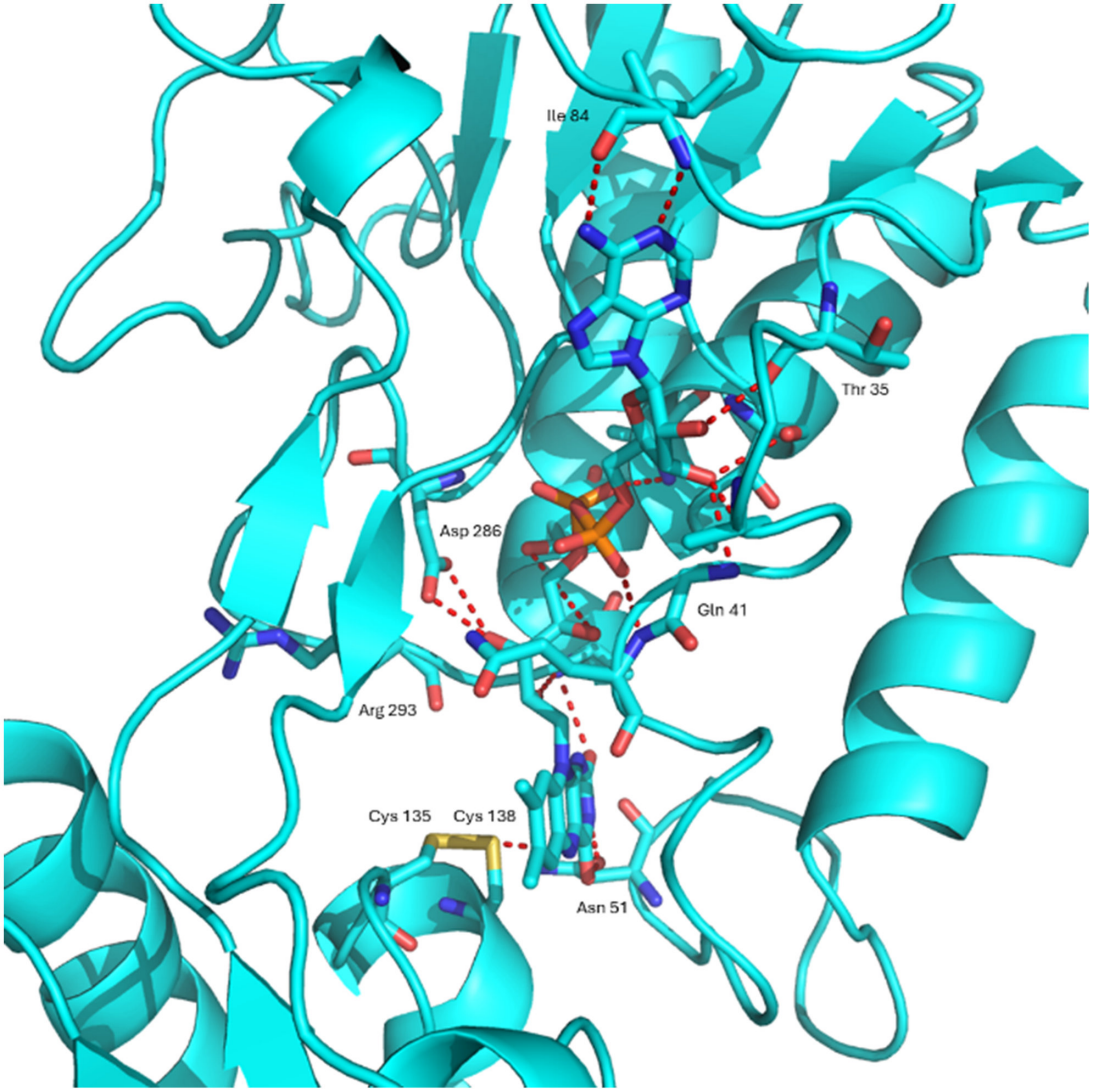
FAD binding site close to the catalytic Cys–Cys motif at the interface of the two domains of one of the Bc‐TrxR subunits.

Interestingly, inspection of the difference Fourier electron density maps reveals the presence of the FAD cofactor in the FAD domain, close to the catalytically important cysteines (Cys135‐Cys138), which are oxidized in our structure, as observed in the structure of *E. coli* reported in the PDB under the accession code 1TDE [18] (Figure 6). The catalytic Cys–Cys motif is located within the redox‐active site at the interface of the two domains, in a position compatible with catalytic turnover [15]. Cys135 is almost totally buried in the X‐ray structure of the FAD‐bound form of the enzyme while Cys138 has SG atom with a reduced accessibility. This suggests that Cys138 could be the most likely target of gold compounds. These observations provide a structural rationale for potential coordination of thiophilic metal ions when cysteines are reduced. Importantly, the availability of the Bc‐TrxR structure establishes a structural framework for interpreting metallodrug binding events observed in solution and for rationalizing enzyme inhibition at the molecular level.

### 2.3 Electrospray Ionization Mass Spectrometry Characterization of the Native Enzyme

**FIGURE 7:**
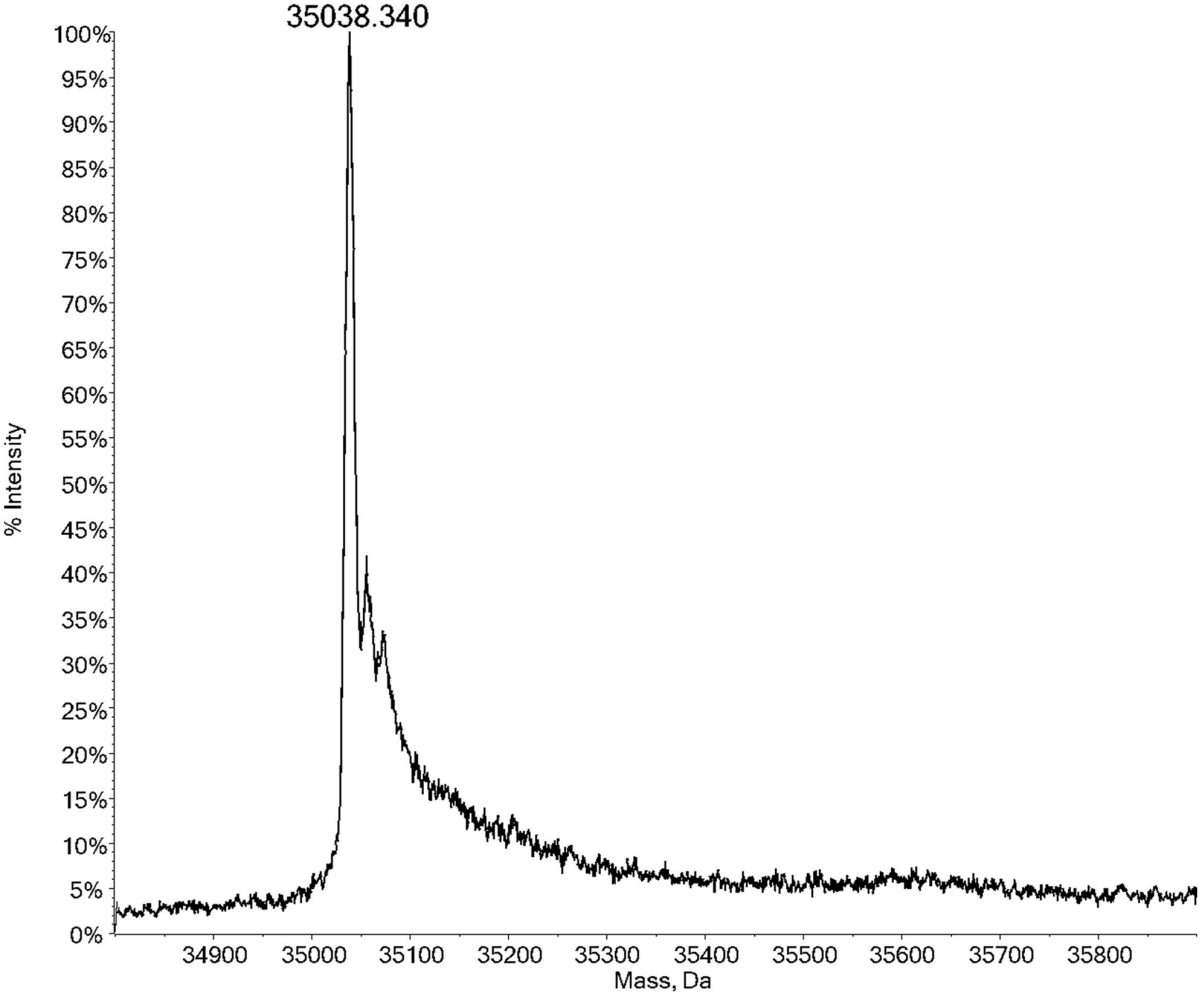
Deconvoluted +TOF LC‐ESI‐MS spectrum of recombinant Bc‐TrxR bearing a C‐terminal 6×His‐tag, recorded at 10^−5^ M (monomer concentration) in 20 mM Tris‐HCl pH 7.5, 150 mM NaCl, and 1 mM DTT, in the m/z 400–3000 range (8.661–8.696 min; isotope resolution 30,000).

ESI‐MS is one of the most powerful techniques for characterizing large biomolecules in solution. High‐resolution ESI‐MS analysis of recombinant Bc‐TrxR under native‐like conditions yielded a molecular mass of 35,038 Da, which is nicely consistent with the theoretical value of 35,041 Da derived from the primary sequence 10–326, with no evidence of truncation or degradation (Figure 7). As the protein is homodimeric and undergoes monomerization during ionization, the observed mass corresponds to that of the monomeric species. As anticipated, the two cofactors, NADPH and FAD, are lost during ionization and their mass is not included in the calculation.

Taken together, the results of the preliminary characterization carried out on recombinant Bc‐TrxR through various methods demonstrate that the protein is properly folded, cofactor‐loaded, structurally intact, and thermostable, thus providing a reliable system for further studies.

### 2.4 ESI MS Studies of Gold Binding

**FIGURE 8:**
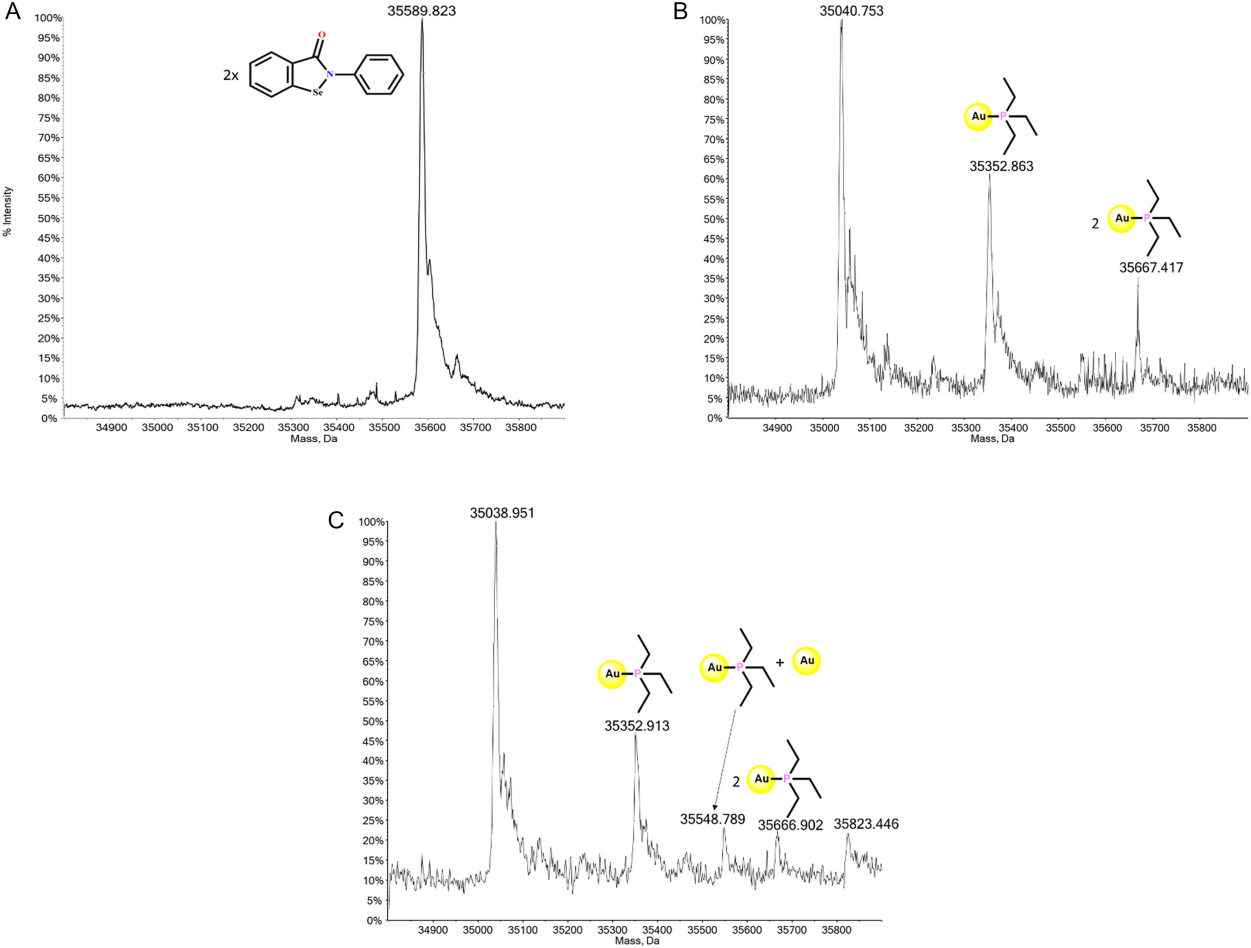
Deconvoluted +TOF LC‐ESI‐MS spectrum of Bc‐TrxR incubated with ebselen at 1:4 protein to ebselen molar ratio at *t* = 30 min (A), and AF (1:3 protein to gold compound molar ratio, 3 h incubation) (B,C) (1:3 protein to gold compound molar ratio, 24 h incubation). Protein concentration: 10^−5^ M protein in 20 mM Tris‐HCl pH 7.5, 150 mM NaCl, 1 mM DTT. Spectra acquired in the m/z 400–3000 range (8.661–8.696 min; isotope resolution: 30,000).

ESI‐MS measurements were then carried out to investigate the interactions of the enzyme with the aforementioned gold compounds (**AF**, **Au1,** and **Au2**). First, the enzyme was reacted with ebselen, an organoselenium compound that selectively targets accessible cysteines. After treating the enzyme with a fivefold excess of ebselen, the spectrum shown in Figure 8A was recorded. This spectrum indicates that the enzyme can bind two ebselen equivalents per Bc‐TrxR subunit, suggesting that two cysteines are selectively modified. Notably, the conversion of the enzyme to its ebselen‐bound form is rapid and complete. This is evidenced by the appearance of a single new peak corresponding to the ebselen_2_‐Bc‐TrxR subunit adduct, with the concomitant disappearance of the unmodified enzyme signal. This observation suggests that the catalytic cysteines are solvent‐accessible and chemically competent in solution.

The enzyme was then reacted individually with the three gold compounds. For experiments involving **AF** (Figure 8B,C), protein samples were buffer‐exchanged into 20 mM ammonium acetate (pH 6.8) and analysed by direct‐infusion high‐resolution ESI‐MS under native‐like conditions. Spectra acquired after 3 and 24 h of incubation with a threefold molar excess of AF revealed, in the deconvoluted profiles, the coexistence of the free enzyme peak and additional signals corresponding to adducts containing one or two Au(I) fragments derived from the triethylphosphine–gold(I) moiety. The same adduct species were observed at both incubation times, indicating that prolonged incubation does not generate additional detectable protein‐bound gold species. Interestingly, the relative abundance of the metalated forms appears lower after 24 h incubation, suggesting that the initially formed adducts may undergo partial redistribution or reduced stability over time.

**FIGURE 9:**
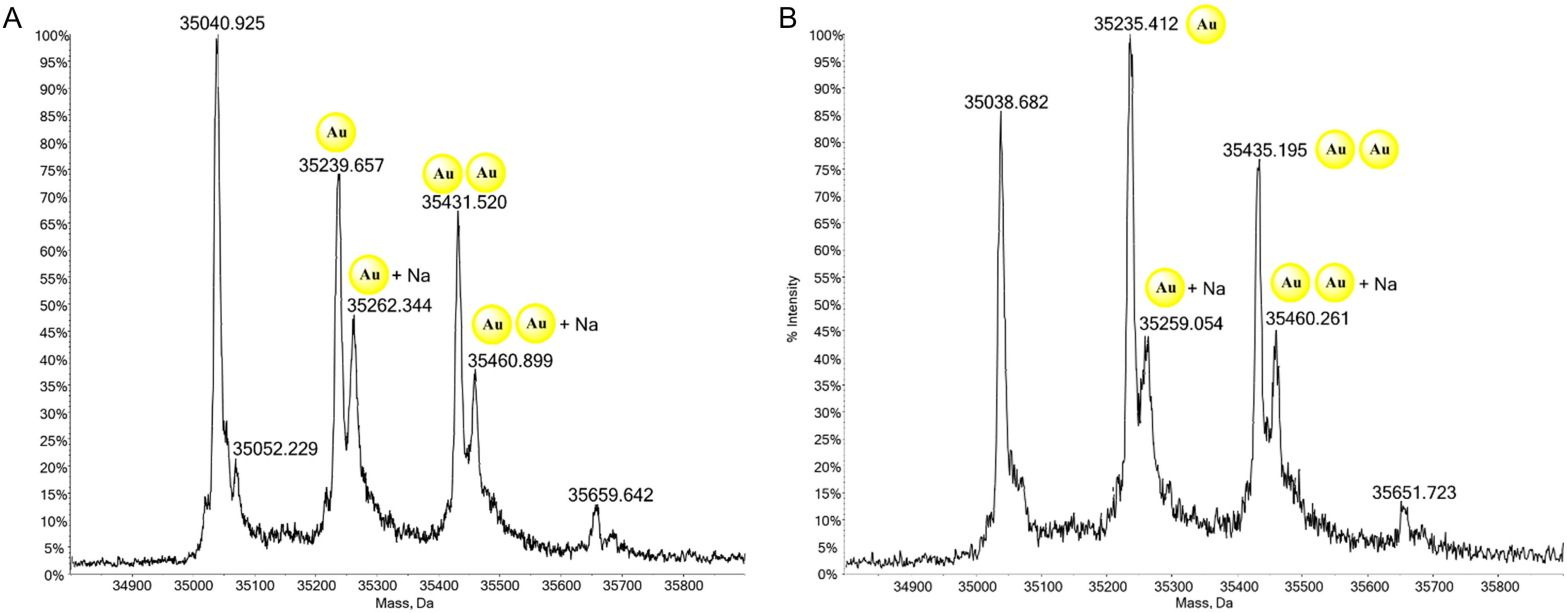
Deconvoluted +TOF LC‐ESI‐MS spectrum of native Bc‐TrxR incubated with **Au1** (A) and with **Au2** (B) (1:3 protein to metal complex molar ratio) for 24 h (10^−5^ M protein) in 20 mM Tris‐HCl pH 7.5, 150 mM NaCl, 1 mM DTT, acquired in the m/z 400–3000 range (8.661–8.696 min; isotope resolution: 30,000).

By contrast, experiments involving **Au1** and **Au2** were performed using LC‐ESI‐MS; samples were kept in a 20 mM Tris‐HCl buffer solution at pH 7.5. We realized that transferring the samples to an ammonium acetate solution reduced the stability of the complexes and their corresponding protein adducts, which is the reason why we used this method. Notably, chromatographic separation improved the resolution of the overlapping species and facilitated the detection of multiple metalated forms. Deconvoluted spectra acquired after a 24‐h incubation period showed three well‐defined peaks corresponding to the free protein, the protein bearing one Au(I) center, and the protein bearing two Au(I) centres (see Figure 9A,B). The same species were observed after a 3h incubation, but with lower signal intensity; these are reported in the Supporting Information (Figure S5).

These spectra suggest the formation of a substantial amount of protein‐gold adducts. Notably, upon protein binding, the complex loses both its original ligands, and the naked gold ion eventually binds to the protein. Similar metalation processes have been extensively reported for mammalian TrxRs, particularly human TrxR1, where auranofin and related gold(I) compounds inhibit enzymatic activity through coordination to catalytically essential selenium‐ and sulfur‐containing residues. Although bacterial low‐molecular‐weight TrxRs lack the C‐terminal selenocysteine motif characteristic of mammalian enzymes and instead contain a redox‐active Cys–Cys pair, both enzyme classes appear susceptible to inhibition through the strong affinity of Au(I) for catalytically relevant chalcogen atoms. Accordingly, the gold–enzyme adducts detected here provide direct molecular evidence that a similar metalation‐based inhibition mechanism operates in Bc‐TrxR [19, 20]. As shown in previous papers, gold(I) binding is selective for free cysteines; moreover, this behaviour is reminiscent of that observed upon reacting *Cryptosporidium parvum* TrxR with a variety of gold compounds (Angelucci et al., Manuscript in preparation).

## 3 Materials and Methods

### 3.1 The Gold(I) Compounds

The structure of the gold compounds (**AF**, **Au1,** and **Au2**) used in this study is shown in Figure 1. AF was purchased from Sigma–Aldrich (Missouri, USA). **AF.** Elemental Analysis calculated for C_20_H_34_AuO_9_PS: C, 35.72; H, 5.01; S, 4.72. Found: C, 35.42; H, 4.89; S, 4.73. ESI‐MS (+): m/z calcd for [C_20_H_34_AuO_9_PS]^+^ 679.1399; found 679.1357.

**Au1** and **Au2** were synthesized according to methodologies reported in the literature [21]. **Au1.** Yield (42%). Elemental Analysis calculated for C_6_H_16_AuOPS: C, 20.79; H, 4.60; S, 10.04. Found: C, 20.65; H, 4.46; S, 9.98. ^1^H NMR (400 MHz, DMSO‐d6) δ 4.33 (t, 2H), 3.50 (m, 2H), 2.77 (m, 2H), 1.54 (m, 9H) ppm. ^31^P{^1^H} NMR (162 MHz, DMSO‐d6) δ 0.92 ppm. ESI‐MS (+): m/z calcd for [C_6_H_16_AuOPS + H]^+^ 365.0398; found 365.0392.

**Au2.** Yield (62%) Elemental Analysis calculated for C_5_H_14_AuOPS: C, 17.44; H, 4.25; S, 9.84. Found: C, 16.94; H, 3.87; S, 10.35. ^1^H NMR (400 MHz, DMSO‐d6) δ 4.62 (t, 2H), 3.43 (m, 2H), 2.77 (m, 2H), 1.54 (m, 9H) ppm. ^31^P{^1^H} NMR (162 MHz, DMSO‐d6) δ 1.02 ppm. ESI‐MS (+): m/z calcd for [C_5_H_14_AuOPS +H]^+^ 351.0247; found 351.0241.

### 3.2 Production and Biochemical Characterization of Recombinant Bc‐TrxR

#### 3.2.1 Expression and Purification of Recombinant Bc‐TrxR

The gene encoding *B. cenocepacia* TrxR (trxB strain J2315) was cloned into the pET29b(+) vector bearing a C‐terminal His_6_‐tag (Twist Bioscience, San Francisco, USA). The plasmid was transformed into *E. coli* BL21(DE3) competent cells.

Transformed cells were grown in Luria–Bertani (LB) medium supplemented with kanamycin (50 μg mL^−1^) at 37°C with shaking (4.5 × g). At OD_600_ 0.6–0.8, protein expression was induced by addition of 1 mM IPTG, followed by incubation at 30°C for 3 h. Cells were harvested by centrifugation and resuspended in lysis buffer (50 mM Tris‐HCl, pH 7.5, 500 mM NaCl, 20 mM imidazole). Cell disruption was achieved by sonication on ice. The lysate was clarified by ultracentrifugation (1.7 × 10^5^ × g, 40 min, 4°C). The supernatant was loaded onto a HisTrap HP 5 mL Ni^2+^‐affinity column (Cytiva) equilibrated with lysis buffer. The protein was eluted using a linear gradient of imidazole (20–500 mM). Fractions containing Bc‐TrxR were pooled and further purified by size‐exclusion chromatography (HiLoad 16/600 Superdex 75 column) equilibrated in 20 mM Tris‐HCl, pH 7.5, 150 mM NaCl, 1 mM dithiothreitol (DTT). Protein purity was assessed by SDS‐PAGE. Concentration was determined spectrophotometrically at 280 nm, using a molar extinction coefficient of 24 410 M^−1^ cm^−1^ (M^−1^ cm^−1^).

#### 3.2.2 UV–Visible Absorption Spectroscopy

UV–visible absorption spectra were recorded at room temperature using a quartz cuvette (1 cm path length) on a UV–vis spectrophotometer. Measurements were carried out in 20 mM Tris‐HCl buffer (pH 7.5) containing 150 mM NaCl. Spectra were collected in the 250–600 nm range. The characteristic absorption bands of the FAD cofactor were monitored to confirm its incorporation into the purified enzyme, with typical maxima observed at ≈380 and 460–490 nm. Protein concentration was estimated from the absorbance at 280 nm using the Beer–Lambert law and the calculated molar extinction coefficient for Bc‐TrxR (ε280 = 24,410 M^−1^ cm^−1^).

#### 3.2.3 Circular Dichroism Spectroscopy

Far‐UV CD spectra were recorded at 25°C using a quartz cuvette with a 0.1 cm path length. Measurements were performed in 5 mM sodium phosphate buffer (pH 7.5) after buffer exchange of the purified protein. Spectra were collected between 190 and 250 nm, averaged over multiple scans, and corrected by subtracting the buffer baseline. The protein concentration used for CD measurements was 0.132 µM. Secondary structure content was estimated using the BeStSel online analysis tool [22]. Thermal denaturation experiments were performed by monitoring the ellipticity at 222 nm while increasing the temperature at a constant rate. Thermal unfolding was followed over the temperature range 20–90°C with a heating rate of 1°C min^−1^. The melting temperature (*T*_m_) was estimated from the midpoint of the unfolding transition. All measurements were performed in triplicate.

#### 3.2.4 Electrospray Ionization Mass Spectrometry

Protein samples were prepared for mass spectrometric analysis according to the compound under investigation. For experiments involving ebselen and AF, samples were buffer‐exchanged into 20 mM ammonium acetate (NH_4_OAc), pH 6.8, and analysed by direct infusion high‐resolution ESI‐MS in positive ion mode to preserve native‐like conditions. In contrast, for experiments involving **Au1** and **Au2**, samples were maintained in 20 mM Tris‐HCl buffer pH 7.5, as these metal complexes displayed reduced stability and/or solubility upon transfer to ammonium acetate; under these conditions, Tris‐HCl ensured preservation of the protein–metal adducts prior to ionization without detectable buffer‐related adduct formation or signal suppression.

For adduct formation experiments, recombinant Bc‐TrxR was incubated with the selected compounds at defined molar ratios (typically 1:1 to 1:3 protein: compound) for 3 and 24 h at 37°C under the buffer conditions specified for each experiment (e.g., 20 mM Tris, 150 mM NaCl, 1 mM DTT, or 5 mM sodium phosphate, depending on sample preparation). In selected experiments, Auranofin was incubated with Bc‐TrxR at a 1:3 molar ratio, while **Au1** and **Au2** were tested under analogous conditions. In complementary experiments, ebselen was incubated with Bc‐TrxR following the same protocol to evaluate adduct formation. Following incubation, samples were analysed by high‐resolution LC–ESI‐MS or direct infusion ESI‐MS, according to the conditions described above. Spectra were acquired in positive ion mode over the specified m/z range, and raw data were deconvoluted using standard software (isotope resolution set to 30,000) to obtain zero‐charge mass distributions. Mass shifts relative to metal‐free Bc‐TrxR were determined from the deconvoluted spectra and assigned to protein–compound adducts based on the observed mass increments.

### 3.3 Crystallization and Structure Determination

#### 3.3.1 Protein Crystallization

Purified Bc‐TrxR was concentrated to 45 mg mL^−1^ (1.3 mM) in 20 mM Tris‐HCl at pH 7.5. Initial crystallization screening was manually performed using the hanging drop vapor diffusion and selected conditions from Kit1, Kit2, and Kit Index by Hampton Research. Best Bc‐TrxR crystals were obtained at 20°C using a protein concentration of 31 mg mL^−1^ (0.9 mM) in 20 mM Tris‐HCl at pH 7.5 and a reservoir containing 2.0 M ammonium sulphate, 0.1M Tris‐HCl pH 8.5. Before freezing, crystals were soaked in a cryoprotectant solution (25% glycerol, 2.0 M ammonium sulphate, 0.1M Tris‐HCl pH 8.5).

#### 3.3.2 X‐Ray Diffraction Data Collection, Structure Resolution, and Refinement

Diffraction data were collected on frozen crystals at 100 K at XRD2 beamline of Elettra synchrotron in Trieste, Italy and processed using Autoproc [23] (see Table S1 for data collection statistics). The structure was solved by molecular replacement using the PHASER software implemented [24] in the CCP4 package and the structure of TrxR from *E. coli* deposited in the PDB under the accession code 1TDE [14] as a starting model. The structure was isotropically refined using REFMAC5 [25]. Visual inspection of the electron density maps and model building were carried out using Coot [26]. Figures were prepared using PyMOL (www.pymol.org). Structure factors and coordinates were deposited in the PDB under the accession code 31LC.

## 4 Conclusion

TrxR has emerged as a key molecular target underlying the antibacterial activity of gold(I) compounds against the opportunistic pathogen *B. cenocepacia*. To gain further insight into how gold compounds cause enzyme inhibition, we produced recombinant Bc‐TrxR in high yield and determined its crystal structure, which provides a structural framework for mechanistic interpretation. The good correspondence between the crystal structure and the solution structure of the protein was confirmed. Then, the interaction of Bc‐TrxR with three selected gold compounds, namely **AF**, **Au1,** and **Au2**, which were previously identified as potent inhibitors of the enzyme, was investigated using high‐resolution ESI‐MS. These studies clearly demonstrate the formation of stable gold–enzyme adducts in solution. Detailed mass spectrometric analysis revealed the presence of metalated protein species consistent with the coordination of either naked gold(I) ions or gold(I)‐containing molecular fragments to the enzyme. The nature of the protein‐bound metallic fragment could be ascertained on a case‐by‐case basis. Overall, our results suggest that the potent inhibition of Bc‐TrxR catalytic activity arises from the direct coordination of gold ions to the catalytically relevant cysteine residues within the active site. The strong thiophilicity of Au(I) and the formation of stable Au–thiolate bonds provide a clear molecular rationale for enzyme inactivation. These findings clarify the biochemical basis of gold‐mediated antibacterial activity and support the rational design of improved, thiol‐targeting gold agents for treating infections caused by *B. cenocepacia*. While the present structural and mass spectrometric data strongly support a cysteine‐targeting inhibition mechanism, future site‐directed mutagenesis studies of the catalytic Cys135/Cys138 residues will be instrumental in establishing their precise contribution to gold coordination and enzyme inactivation.

## Funding

This study was supported by MIUR PRIN 2022 (2022JMFC3X).

## Conflicts of Interest

The authors declare no conflicts of interest.

## Supporting information

The authors have cited additional references within the Supporting Information.

## Data Availability

The atomic coordinates and structure factors for Bc‐TrxR have been deposited in the Protein Data Bank (PDB) under accession code 31LC. The corresponding DOI is: https://doi.org/10.2210/pdb_000031lc/pdb.
