# Supplementary material for: Thioredoxin Reductase as a Target for Antibacterial Gold Compounds in Burkholderia cenocepacia: Disclosing the Molecular Basis of Enzyme Inhibition
Source: Chembiochem. 2026 Jul 10;27(13):e70462. doi: 10.1002/cbic.70462 (PMC13351807; doi:10.1002/cbic.70462)
Supplement: Supplementary file 1 — The authors have cited additional references within the Supporting Information. [file CBIC-27-e70462-s001.pdf]

Supplementary Materials

Thioredoxin Reductase as a Target for Antibacterial Gold Compounds in *Burkholderia Cenocepacia*: Disclosing the Molecular Basis of Enzyme Inhibition

Stefano Zineddu[a#], José Aleixo de Azevedo-França[a*#], Martina Aguanno[a], Valentina Pecchioli[a], Paola Turano[b*], Giarita Ferraro[c], Virginia Cuomo[c], Antonello Merlino[c*] and Luigi Messori[a*]

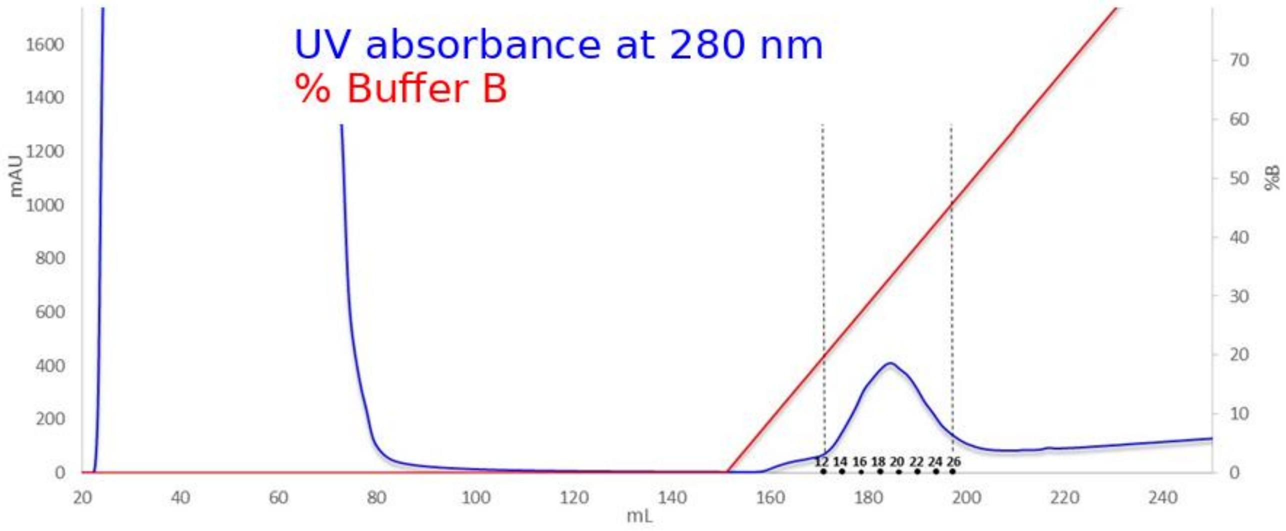

Figure S1. Chromatogram relating to the first step of purification by affinity chromatography. The protein (whose absorbance at 280 nm is shown in blue in the graph) was eluted through a linear gradient of 20-500 mM imidazole in 50 mM TRIS pH 7.5 (%B in red). The fractions are shown in black.

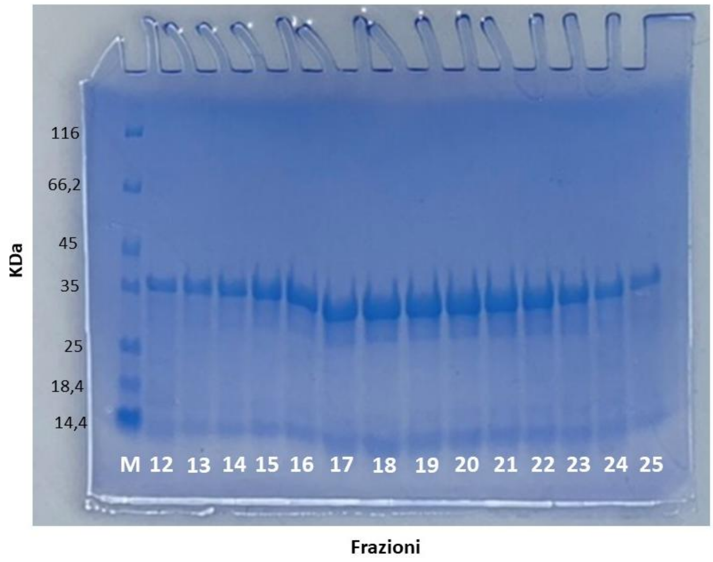

Figure S2. SDS-PAGE gel electrophoresis of fractions 12-25 recovered by affinity chromatography. M = molecular weight marker.

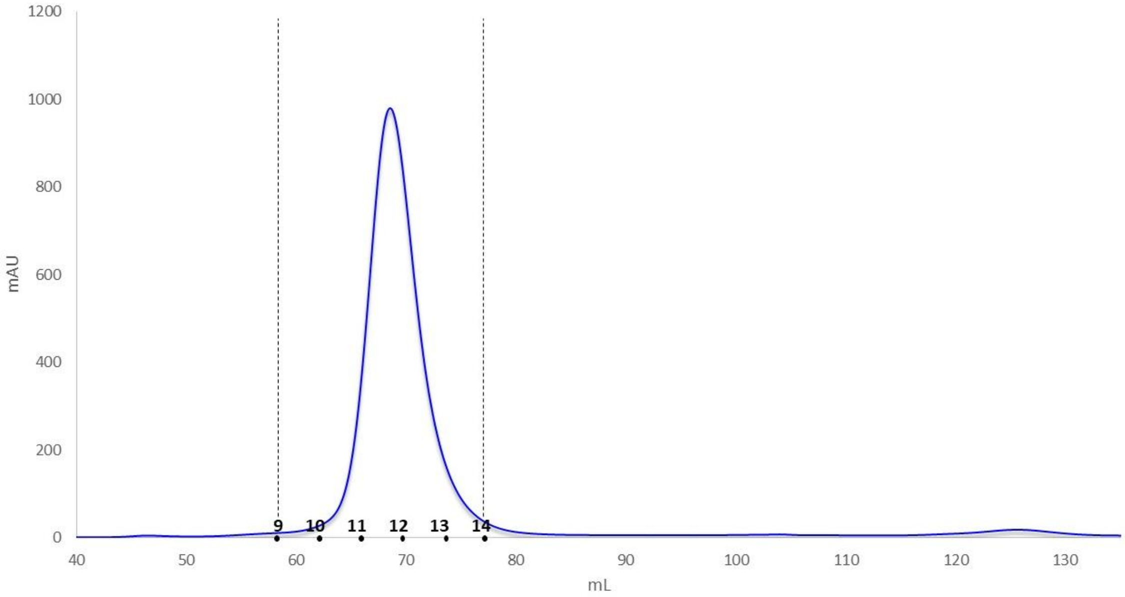

Figure S3. Molecular exclusion chromatogram, relating to the second purification step. The absorbance of the sample at 280 nm is shown in blue and the fractions are represented in black.

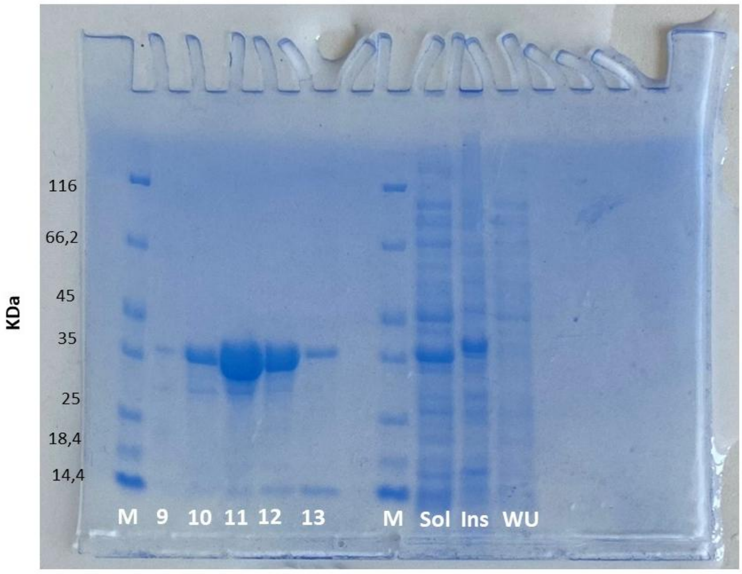

Figure S4. SDS-PAGE gel electrophoresis of fractions 9-13 recovered by molecular exclusion chromatography, insoluble and soluble fractions saved after ultracentrifugation (Sol and Ins) and unbound wash recovered by affinity chromatography (WU). M = molecular weight marker.

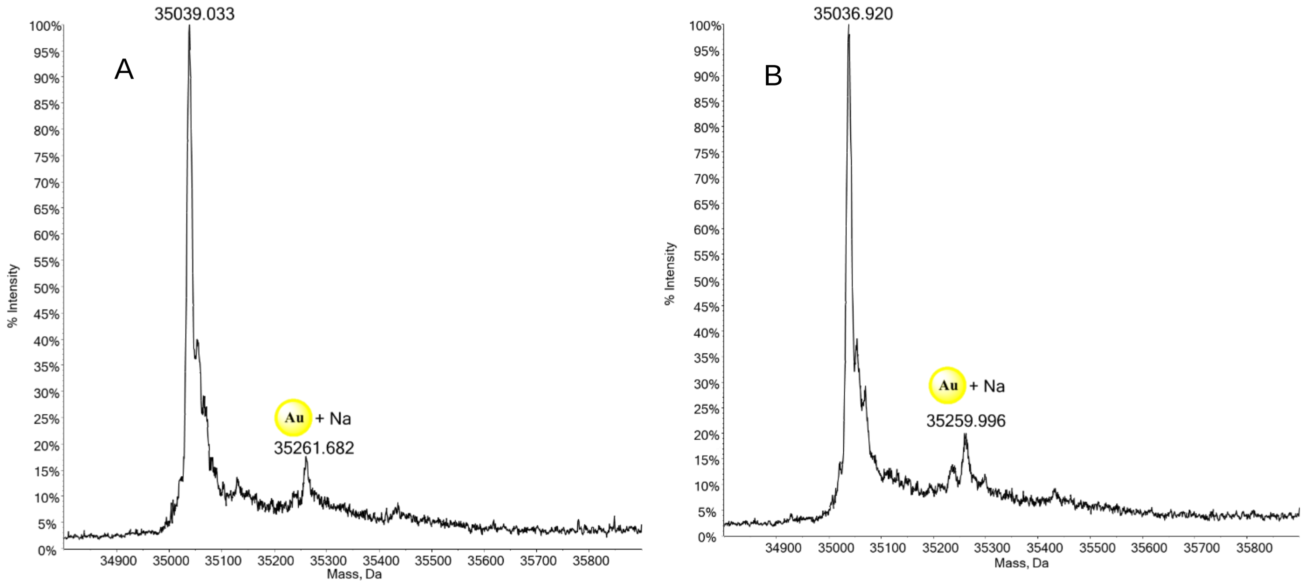

Figure S5. Deconvoluted +TOF LC-ESI-MS spectrum of native Bc-TrxR incubated with **Au1** (A) and **Au2** (B) (1:3 molar ratio) for 3 h (10^{-5} M protein) in 20 mM Tris-HCl pH 7.5, 150 mM NaCl, 1 mM DTT acquired in the m/z 400-3000 range (8.661-8.696 min; isotope resolution: 30,000).

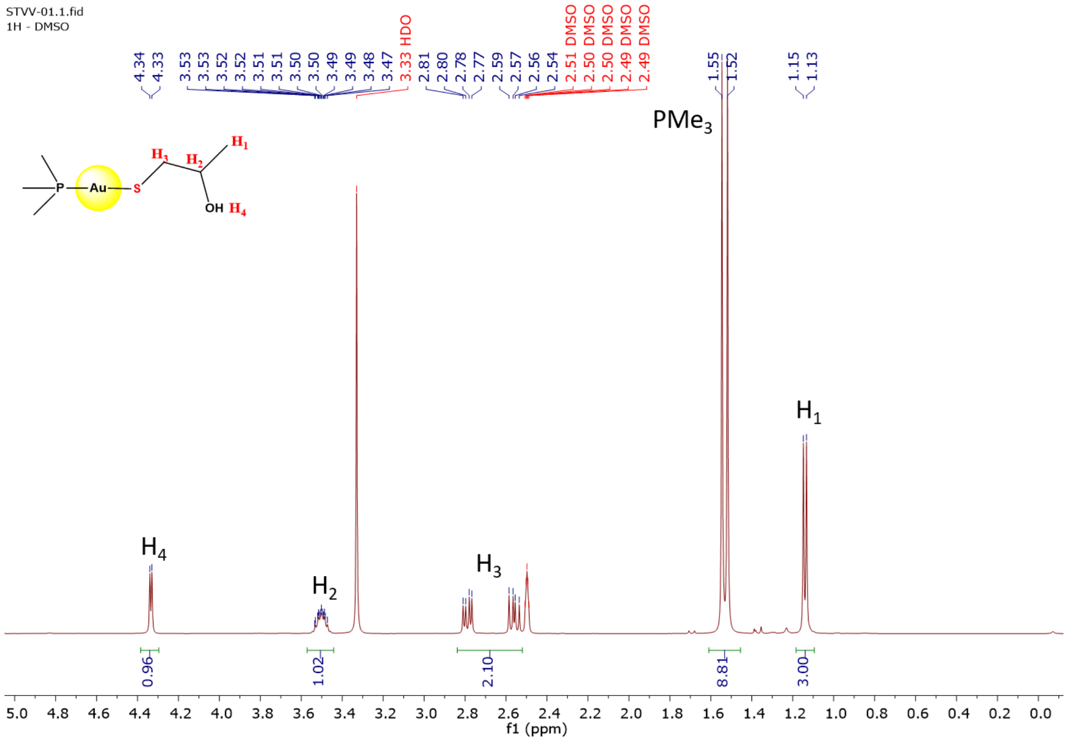

Figure S6. ^{1}H NMR spectrum of Au1 recorded in DMSO-d_6 at 400 MHz.

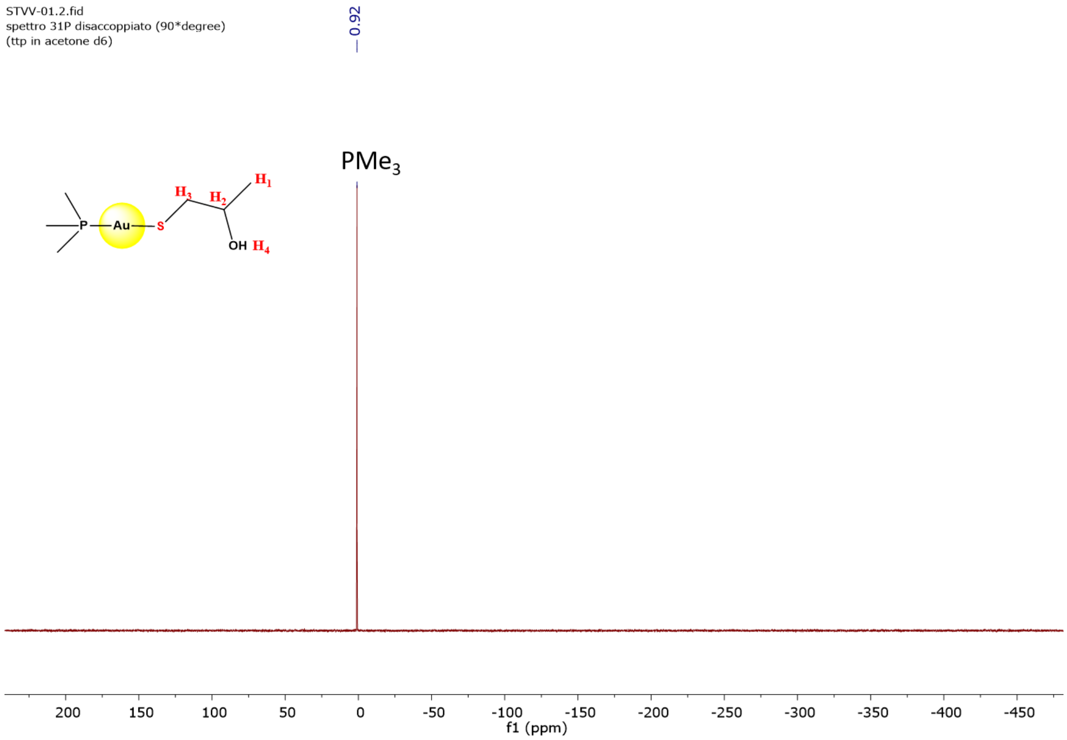

Figure S7. $^{31}P\{^{1}H\}$ NMR spectrum of Au1 recorded in DMSO-d_6 at 162 MHz.

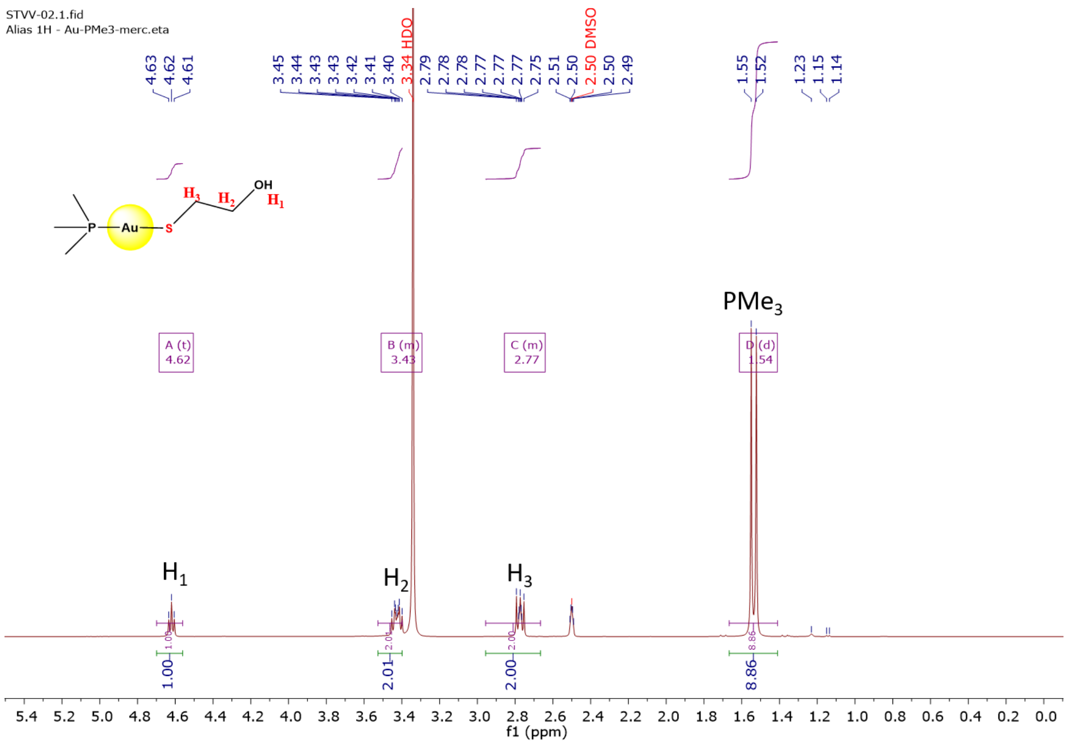

Figure S8. ^{1}H NMR spectrum of Au2 recorded in DMSO-d_6 at 400 MHz.

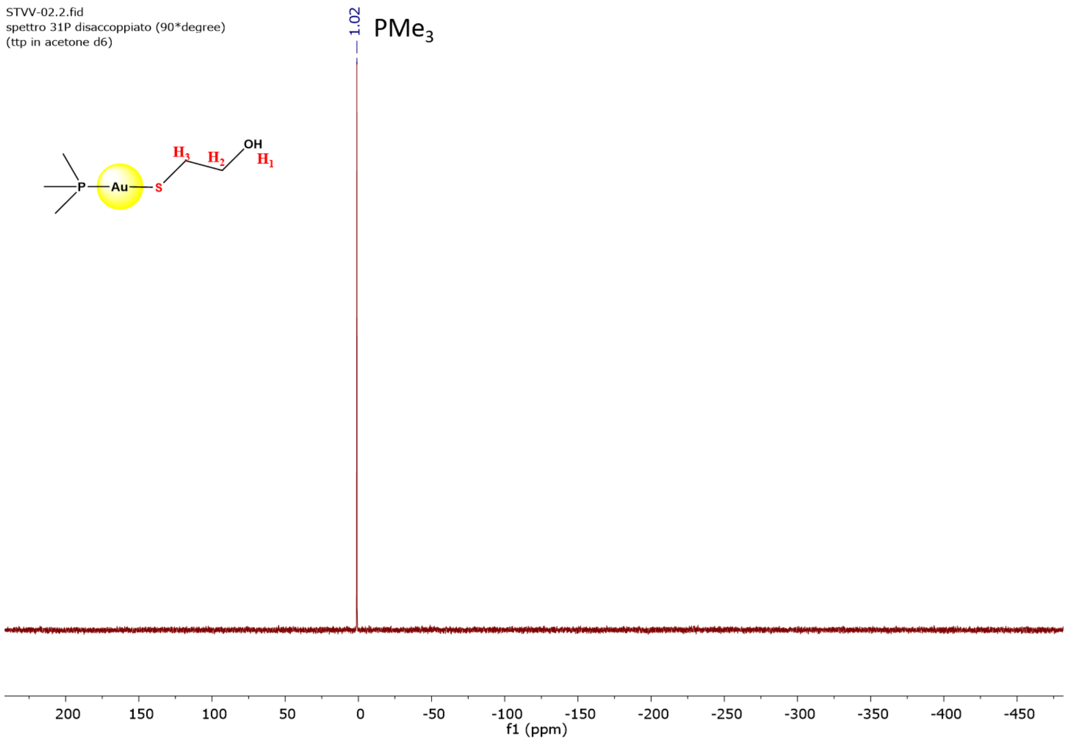

Figure S9. $^{31}P\{^{1}H\}$ NMR spectrum of Au2 recorded in DMSO-d_6 at 162 MHz.

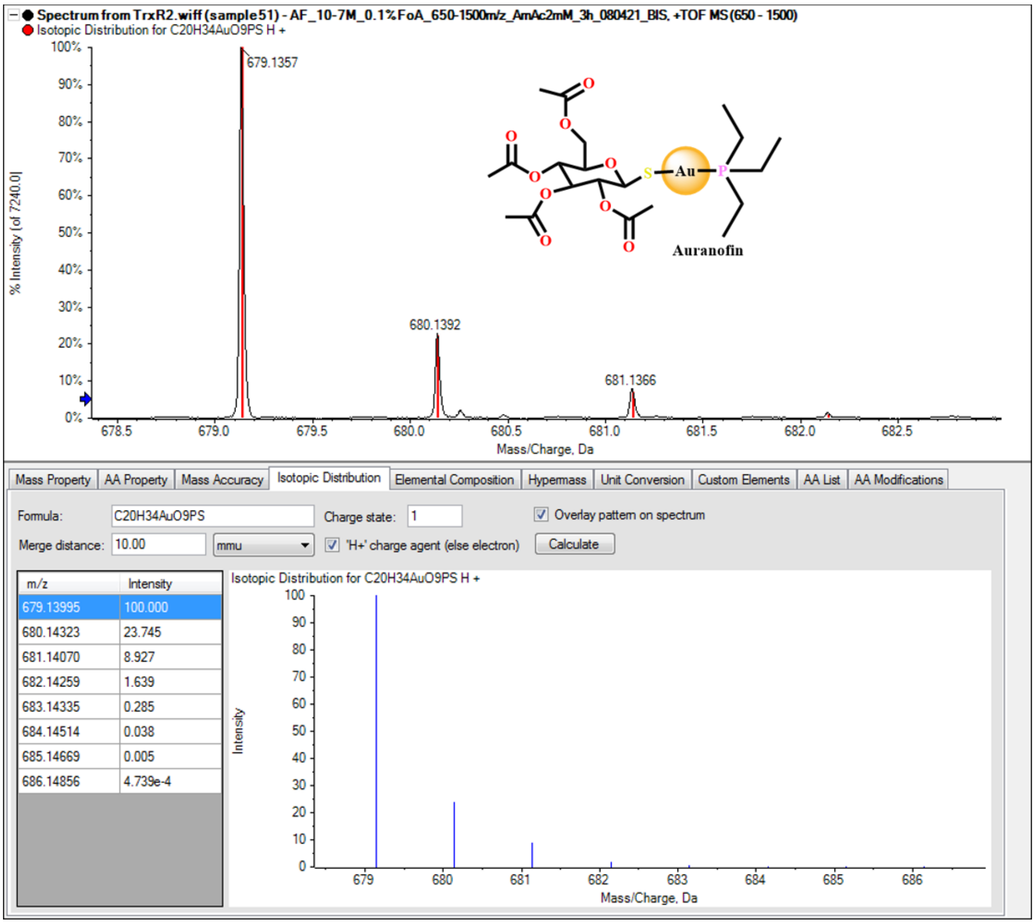

Figure S10. High-resolution ESI mass spectrum of Au1. The experimental isotopic distribution is in good agreement with the theoretical isotopic pattern calculated for the molecular ion $[C20H34AuO9PS]^{+}$ (m/z 679.1399).

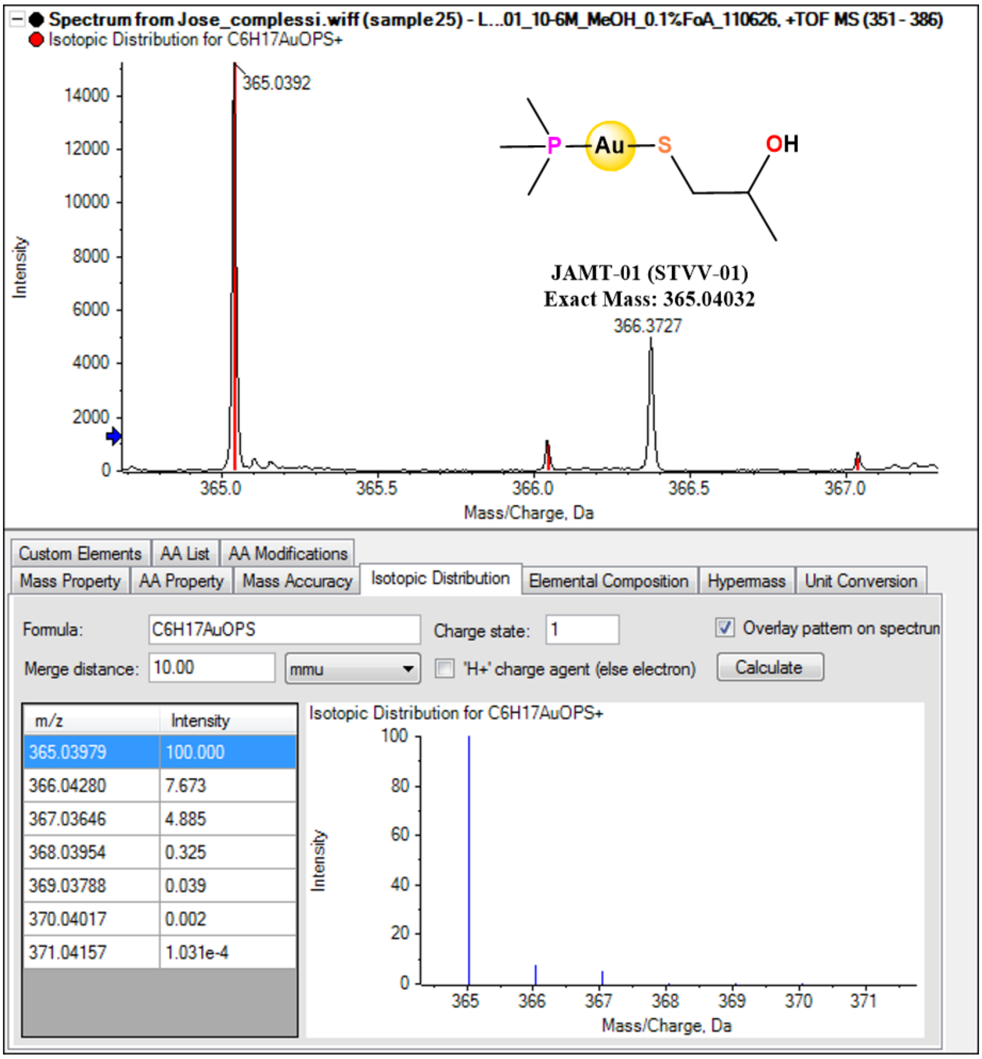

Figure S11. High-resolution ESI mass spectrum of Au1. The experimental isotopic distribution is in good agreement with the theoretical isotopic pattern calculated for the molecular ion $[C_6H_{17}AuOPS]^+$ (m/z 365.0398).

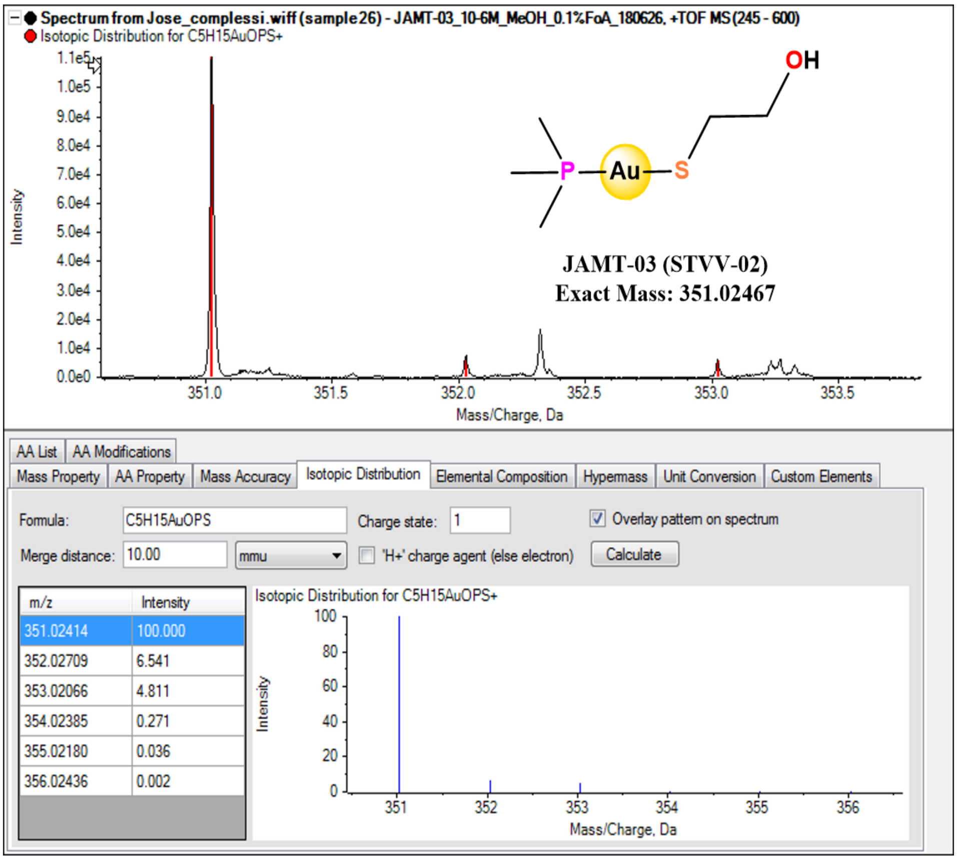

Figure S12. High-resolution ESI mass spectrum of Au1. The experimental isotopic distribution is in good agreement with the theoretical isotopic pattern calculated for the molecular ion $[C5H15AuOPS]^{+}$ (m/z 351.0247).

Table S1. Data collection and refinement statistics

Data collection	
PDB code	31LC
Space group	*P*3_1 *2 1*
a (Å)	68.29
b (Å)	68.29
c (Å)	131.35
α/β/γ (°)	90.00/90.00/120.00
Resolution range (Å)	59.14 - 2.32 (2.36-2.32)
Observations	286641 (15337)
Unique reflections	15470 (795)
Completeness (%)	96.8 (100.0)
Redundancy	18.5 (19.3)
Rmerge (%)	0.074 (6.382)
Average I/σ(I)	22.6 (0.6)
$CC_{1/2}$	1.000 (0.600)
Anom. completeness (%)	96.8 (100.0)
Anom. Multiplicity	10.0 (10.2)
Refinement	
Resolution (Å)	59.14 - 2.52
Reflections	11497
R-factor/R_{free}	26.0/36.6
Non-H atoms in the refinement	2422
Overall B-factor (Å^2)	97.13
R.m.s.d. bonds (Å)	0.006
R.m.s.d. angles (°)	1.654
In favoured regions	225 (82.79%)
Outliers	15 (4.87%)

Table S2. Structural features of oxidized Bc-TrxR and Ec-TrxR monomer

Organism (PDB ID)	Bc-TrxR	Ec-TrxR (oxidized form, PDB code 1TDE)
Helix (%)	26	27
Beta (%)	39	35
Coil (%)	33	37
Turn (%)	12	16
#res with H-bonds	240 (76%)	245 (77%)
Total ASA (Å^2)	15298.6	15358.3
ASA of backbone (Å^2)	1782.7	1939.9
ASA of sidechains (Å^2)	13516.0	13418.4
Exposed nonpolar ASA (Å^2)	8850.5	8432.3
Exposed polar ASA (Å^2)	3237.3	3693.9
Exposed charged ASA (Å^2)	3210.8	3232.1
Side exposed nonpolar ASA (Å^2)	8816.0	8381.9
Side exposed polar ASA (Å^2)	1560.1	1876.5
Side exposed charged ASA (Å^2)	3139.9	3160.0
Fraction nonpolar ASA	0.58	0.55
Fraction polar ASA	0.21	0.24
Fraction charged ASA	0.21	0.21
Mean residue ASA	48.9	48.6
Mean frac ASA	0.3	0.3
% side ASA hydrophobic	24.58	22.89
Total volume (packing) (Å^3)	41017.5	43032.8
Mean residue volume	131.0	136.2

Structural features have been computed using HBAT 2 (https://hbat-web.abhishek-tiwari.com/) and VADAR (https://vadar.wishartlab.com/) Tiwari, A. (2026). HBAT 2: A Python Package to analyse Hydrogen Bonds and Other Non-covalent Interactions in Macromolecular Structures. arXiv. https://doi.org/10.48550/arXiv.2602.17712
